# Construction of a High-Density Genetic Map and Identification of Leaf Trait-Related QTLs in Chinese Bayberry (*Myrica rubra*)

**DOI:** 10.3389/fpls.2021.675855

**Published:** 2021-06-14

**Authors:** Shuwen Zhang, Zheping Yu, Xingjiang Qi, Zhao Wang, Yuanyuan Zheng, Haiying Ren, Senmiao Liang, Xiliang Zheng

**Affiliations:** Institute of Horticulture, Zhejiang Academy of Agricultural Sciences, Hangzhou, China

**Keywords:** *Myrica rubra*, re-sequencing, genetic map, quantitative trait loci mapping, leaf traits

## Abstract

Chinese bayberry (*Myrica rubra*) is an economically important fruit tree that is grown in southern China. Owing to its over 10-year seedling period, the crossbreeding of bayberry is challenging. The characteristics of plant leaves are among the primary factors that control plant architecture and potential yields, making the analysis of leaf trait-related genetic factors crucial to the hybrid breeding of any plant. In the present study, molecular markers associated with leaf traits were identified *via* a whole-genome re-sequencing approach, and a genetic map was thereby constructed. In total, this effort yielded 902.11 Gb of raw data that led to the identification of 2,242,353 single nucleotide polymorphisms (SNPs) in 140 F_1_ individuals and parents (*Myrica rubra* cv. Biqizhong × *Myrica rubra* cv. 2012LXRM). The final genetic map ultimately incorporated 31,431 SNPs in eight linkage groups, spanning 1,351.85 cM. This map was then used to assemble and update previous scaffold genomic data at the chromosomal level. The genome size of *M. rubra* was thereby established to be 275.37 Mb, with 94.98% of sequences being assembled into eight pseudo-chromosomes. Additionally, 18 quantitative trait loci (QTLs) associated with nine leaf and growth-related traits were identified. Two QTL clusters were detected (the LG3 and LG5 clusters). Functional annotations further suggested two chlorophyll content-related candidate genes being identified in the LG5 cluster. Overall, this is the first study on the QTL mapping and identification of loci responsible for the regulation of leaf traits in *M. rubra*, offering an invaluable scientific for future marker-assisted selection breeding and candidate gene analyses.

## Introduction

Chinese bayberry (*Myrica rubra* or *Morella rubra*) is among the most economically important fruit trees grown in southern China. Its fruits are popular for their taste and health benefits, as they are rich in ascorbic acid, phenolic compounds, and anthocyanins, while bayberry leaf extracts are reported to exhibit antiviral, antioxidant, and antimicrobial properties (Chen et al., 2015; Zhang et al., 2015, 2017). Despite their economic value, bayberry trees are challenging to selectively breed, with a prolonged juvenile phase that can require >10 years for trees to initially flower and bear fruit. Breeding plants based on their fruit quality or yield is a particular challenge, and leaf-related traits, such as area and size, are, instead, often used in this context, given that these traits determine the photosynthetic capacity of plants and thereby regulate potential yield (Tang et al., 2018). Leaf color can also similarly impact the yield and quality of different crops (Wang et al., 2019a), in addition to regulating defenses against certain diseases (Demarsy et al., 2018) and controlling anthocyanin biosynthesis (Ullah et al., 2017). It is thus vital that the genetic factors responsible for the regulation of leaf traits in bayberry trees be identified to guide future selective breeding efforts.

Recent advances in genome sequencing technologies have led to the sequencing of apple, pear, kiwifruit, citrus, and pineapple genomes, among others (Velasco et al., 2010; Wu et al., 2012, 2014; Huang et al., 2013; Chen et al., 2019), offering insights into fruit genome evolution and structure, enabling researchers to better guide functional studies and efforts to clone specific genes of interest so as to facilitate molecular breeding. As of 2019, two Chinese bayberry genomes have been published. One is the Zaojia variety, with a predicted genome size of 289.92 Mb genome size and 26,325 putative genes at the scaffold level (Ren et al., 2019), while the other is the Y2012-145 genome, with a predicted size of 313 Mb and 32,493 putative genes arranged into eight pseudo-chromosomes based on a parental genetic linkage map (Jia et al., 2019; Wang et al., 2020). These genomes serve as valuable reference sources for efforts to assess gene functionality and to accelerate bayberry breeding efforts.

The identification of quantitative trait loci (QTLs) associated with particular complex traits is a primary approach to the genetic mapping of plants (Ma et al., 2020; Scheben et al., 2020). Two sets of such genetic maps have been published for *M. rubra* (Jia et al., 2019; Wang et al., 2020), with the first being a biparental genetic linkage map prepared using Join-Map 4.1 based on a 95-member population (Biqi × Dongkui) using SNP markers identified with RAD-Seq and aligned with scaffold and contig sequences. One of these maps contained 3,075 SNP markers aggregated into haplotype blocks (HBs), corresponding to 407 genetic bins, spanning 531 cM and eight linkage groups (Jia et al., 2019). The other set of maps spanned 491 cM and contained 3,191 total markers (3,073 SNPs and 118 SSR markers), with an average interval of 0.15 cM between markers, and was specifically used for sex mapping (Wang et al., 2020). Leaves serve as the primary photosynthetic organs in bayberry trees and many other plants, and the overall morphology of these leaves can thus directly impact light absorption and transmittance to modulate plant yields. Understanding which genes control leaf size is thus invaluable for both selective breeding efforts and for clarifying the mechanisms governing plant developmental biology (Yang et al., 2019). For example, researchers have employed a restriction site-associated DNA sequencing (RAD-seq) approach to develop an integrated *Catalpa bungei* genetic map, containing 9,593 pleiotropic markers across 20 linkage groups, identifying 20 QTLs associated with seven leaf traits and13 QTLs associated with plant height, in addition to predicting that cyclin genes are key determinants of leaf development (Lu et al., 2019). In *Ziziphus jujuba* Mill, a map spanning 2,167.5 cM and containing 3,792 markers across 12 linkage groups was constructed *via* a genotyping-by-sequencing strategy, leading to the identification of 27 leaf trait-related QTLs (Wang et al., 2019b); in *Populus*, a next-generation sequencing (NGS) strategy was used to generate male and female genetic maps, containing 889 and 1,650 SNPs, respectively, that were associated with 42 QTLs linked to nine leaf shape parameters (Xia et al., 2018). In *Citrullus lanatus* L., genome-wide RNA-seq and BSA were combined to identify 5,966 SNPs and indels associated with the lobed leaf trait, enabling the researchers to identify two candidate leaf shape-related genes (Wei et al., 2017). These results underscore the value of genetic mapping as a basis for identifying genes linked to particular phenotypes of interest. These maps can also be leveraged to guide map-based cloning and related efforts, with QTL identification being an essential starting point for the detection of genetic factors associated with particular leaf traits (Kumar et al., 2019; Tahir et al., 2019). Identifying and functionally characterizing QTLs or genes that control leaf shape will further enable the appropriate guidance of future breeding programs and the clarification of the genetic basis for Chinese bayberry leaf morphological and physiological traits.

Herein, we prepared an F_1_ segregating population from the Chinese bayberry Biqizhong (female) and 2012LXRM (male) parental cultivars and then employed a whole-genome resequencing technology-based approach to construct a high-density genetic map. We then identified key QTLs associated with leaf and plant growth traits and assembled the previously published scaffold genome (Ren et al., 2019) into eight pseudo-chromosomes based on this map. This study is the first QTL-based study of plant and leaf growth traits in Chinese bayberry to our knowledge, laying a foundation for future functional genetic studies and for marker-assisted selection (MAS) efforts.

## Materials and Methods

### Materials and Nucleic Acid Extraction

A female Biqizhong plant was selected for the present study, as this cultivar is the most representative Chinese bayberry variety with a wide planting range, a long history of cultivation, and robust adaptability. Male 2012LXRM plants with red flowers were additionally selected based on their long flowering period and large pollen quantities. In 2012, pollen was collected from these two parental plants (both 20 years old) for hybridization, and the flow chart of F_1_ population construction was shown in Supplementary Figure 1. About 1,500 F_1_ progeny seeds were sown and grown in the greenhouse of the Zhejiang Academy of Agricultural Sciences. In 2015, all F_1_ individuals were transplanted to an experimental field, with a 1.2 m × 1.2 m spacing scheme between trees. Young healthy leaf samples from 140 F_1_ individuals and from both parental plants were then collected in September 2019 and were snap-frozen prior to storage at −80°C. A modified cetyltrimethylammonium bromide (CTAB) approach was then used to prepare DNA from these leaves (Jia et al., 2015).

In May 2020, young and fresh leaves and south-facing annual branches positioned 1 m above the ground were collected from parental plants for transcriptomic sequencing, with three replicates per tree being prepared. Trizol (Invitrogen) was used to isolate RNA from these samples based on provided protocols, with an Agilent 2,100 Bioanalyzer (Agilent Technologies, Inc., CA, United States) being used to assess RNA quality and quantity.

### Phenotypic Analyses

F_1_ plant leaf parameters were assessed in May 2020, with analyses being specifically conducted using 2nd-year leaves on the first-order branches of the main stem. Leaf length (LL), leaf width (LW), and leaf area (LA) were detected *via* a leaf area meter (YMJ-B). The LW ratio was calculated by LL/LW. Leaf perimeter (LP) and trunk perimeter (TP) were measured using a flexible ruler. Leaf thickness (LT) was determined using Vernier calipers for five leaves. Chlorophyll content (SPAD) was measured at three different positions per leaf with a SPAD-502 Plus chlorophyll meter (Konica Minolta Holdings, Inc., Chiyoda-ku, Tokyo, Japan). Plant height (PH) was the distance from the root to the top of the plant, measured with a flexible ruler. Individual analyses were repeated 30 times.

### Library Construction, Re-sequencing, and Transcriptome Sequencing

DNA degradation for all 142 samples was initially assessed using 1% agarose gels, while a Qubit® DNA Assay Kit in Qubit® 3.0 Fluorometer (Invitrogen, United States) was used to assess DNA concentrations in these samples. Next, a Covaris S220 instrument (Covaris, United States) was used to prepare ~350 bp fragments from 1 μg of DNA per sample based on the provided directions. Adapters were then ligated to 3′-fragment ends. These ligated adapters were then amplified *via* PCR, purified, and the resultant libraries were sequenced with an Illumina HiSeq X-10 instrument (Illumina, United States) *via* a 150 bp paired-end read approach (Liu et al., 2019; Zhang et al., 2019).

A total of six RNA-seq libraries were prepared and assessed using an Illumina HiSeq 2,500 instrument (Illumina). Low-quality reads (>10% Ns or a PHRED score <10 for at least 50% of bases), and those containing adaptor sequences were removed from the resultant reads using an internally developed script. The expression levels of mapped-paired reads were normalized as reads per kilobase per million mapped reads (RPKM), with the CLC RNA-Seq analysis tool (Adal et al., 2021). The normalized reads were then used for the differential expression analyses in two groups. Differentially expressed genes were identified based on criteria set as an absolute log 2-fold change ≥1, and a false discovery rate (FDR) value of *p* ≤ 0.05. The Mercator4 vs. 2.0 platform (Schwacke et al., 2019) was employed to annotate the differentially expressed gene sequences with default settings. Heatmap cluster analysis was performed for these genes, with the MeV 4.9.0 program (Howe et al., 2011).

### Single Nucleotide Polymorphism Identification, Genotyping, and Validation

A DNA library was prepared using gDNA from parents and 140 F_1_ individuals, and was sequenced using an Illumina HiSeq2500 instrument. Alignment of these reads with a reference genome (Ren et al., 2019) was then conducted with the Burrows-Wheeler Aligner (BWA, mem -t 4 -k 32 -M -R) tool, as it can facilitate the alignment of low-divergence sequences with large reference genomes (Li and Durbin, 2010). Alignment files were converted into BAM files with the SAM tools program (Li et al., 2009), and SNPs were identified using GATK (4.1.3) based on the following criteria: parents should not have <11 base support numbers, and offspring should not have < five base support numbers, and the quality of the variation should not be smaller than 60. After hard filtering, a custom Perl script was used to filter SNPs (only exhibiting segregation X^2^ < 0.00001 are included), and SNP markers with more than 10% of missing data or duplicated markers (markers with the same genotype for all individuals) were discarded. The variant effects of identified high-quality SNPs were predicted using an ANNOVAR approach (Wang et al., 2010).

KASP marker design was based on SNPs within 50 bp upstream and downstream regions as determined using the Cereals DB website^1^ (Wilkinson et al., 2016). SNP accuracy was validated using KASP assays designed based on corresponding read sequences, harboring SNPs of interest mapped to QTL regions. For these assays, a 1.6 μl PCR reaction system, containing 0.8 μl of KASP Master mix (LGC, Biosearch Technologies), 0.05 μl of each primer, and 0.8 μl of DNA (5–10 ng/μl), was analyzed *via* PCR based on provided directions with an IntelliQube instrument (LGC, Biosearch Technologies). KASP-SNPs were compared with corresponding SNPs to establish numbers of mismatches, and converted KASP-SNPs were validated on a population of 50 F_1_ individuals.

### Genetic and Physical Map Construction and Comparative Analysis

The Lep-MAP3 software^2^ was used for genetic map construction based on the maximum-likelihood method (Rastas, 2017). Filtered markers with LOD values of different gradients were clustered where possible, with optimal clustering being achieved at a LOD = 7, yielding eight major linkage groups that corresponded well with available chromosome-related information. Genetic distances and the sorting of markers in each group were calculated *via* the Kosambi algorithm.

Marker physical and genetic positions were used to orient previously published scaffolds (Diop et al., 2020), with a physical chromosome map then being constructed based on the integrated linkage map. Scaffolds with more than two SNPs were oriented based on the most common orientation as determined based on all possible mapped marker pairs, which consider potential orientations within the integrated genetic map. Genome alignment was then used to further order and extend short and adjacent scaffolds based on homologous regions, with adjacent scaffolds on separate chromosomes being separated by 1,000 N’s. The Python-based MCscan (Wang et al., 2012b; Chalhoub et al., 2014) was used when analyzing synteny and collinearity between the Chinese bayberry genome and the *Jugians regia* and *Medicago sativa* genomes.

### Quantitative Trait Loci Mapping, Candidate Gene Selection, and qPCR

Average F_1_ individual values were used for QTL analyses conducted *via* composite interval mapping (CIM) in Windows QTL Cartographer 2.5 (Wang et al., 2012a; Zeng et al., 2019). QTLs with LOD values of 2–3 were identified as potential QTLs (Lander and Kruglyak, 1995), and QTLs with LOD values at or above a threshold value defined through a permutation test (1,000 repeats) were ultimately defined as QTLs (Churchill and Doerge, 1994). Overlapping regions detected *via* both approaches were considered to correspond to confidence intervals. QTL effects were estimated based on the proportion of phenotypic variance explained (PVE) by a given QTL, and QTL naming was conducted a “q,” followed by an abbreviation of the trait name and a serial number to distinguish different loci of the same trait (Zhao et al., 2021). All genes in QTL clusters were detected and categorized as per GO, KEGG, and Nr database annotations. The Zaojia reference genome was used for candidate gene discovery. Genes having key functions related to the investigated traits, encoding ocytochrome, chlorophyll, and growth-regulation-associated proteins and located within the QTL clusters, were considered as candidate genes (Zhang et al., 2021).

A FastKing RT Kit with gDNase (KR180123) was used to prepare cDNA from RNA samples based on provided directions (TIANGEN Biotech Co., Ltd., Beijing, China); after which, qPCR was conducted with the SuperRealPreMix Plus kit, including SYBR Green (FP171206; TIANGEN Biotech Co., Ltd.) using a Roche LightCycler 96 instrument (Roche Molecular Systems, Inc., CA, United States). Each reaction was conducted in a 20-μl volume, containing 2x SuperRealPreMix Plus (10 μl), 0.6 μl of each primer, cDNA (2 μl), and RNase-free ddH_2_O (6.8 μl). Thermocycler settings included pre-degeneration (95°C for 30 s, 1 cycle), three-step amplification (95°C for 5 s, 60°C for 30 s, and 72°C for 30 s, 40 cycles), melting (95°C for 1 s, 65°C for 15 s, 95°C for 1 s), and cooling (37°C for 30 s) steps, and gene expression was normalized to that of actin (MrACT, GenBank GQ340770; Niu et al., 2010).

## Results

### Sequencing, Genotyping, and SNP Verification

Following the re-sequencing of 140 F_1_ progeny and their parents, we obtained 902.11 Gb (902, 107, 492, and 200 bp) of raw data. These data were then filtered, yielding 6,014,049,948 clean reads with an average 92.25% Q30 value and an estimated 38.20% GC content. Following mapping to the reference genome, 2,242,353 total SNPs were detected at an average sequencing depth of 22.09 x in the female “Biqizhong” parental plant, 17.00 x in the male 2012LXRM parental plant, and 16.92 x in each F_1_ offspring (Supplementary Table 1).

**Figure 1:**
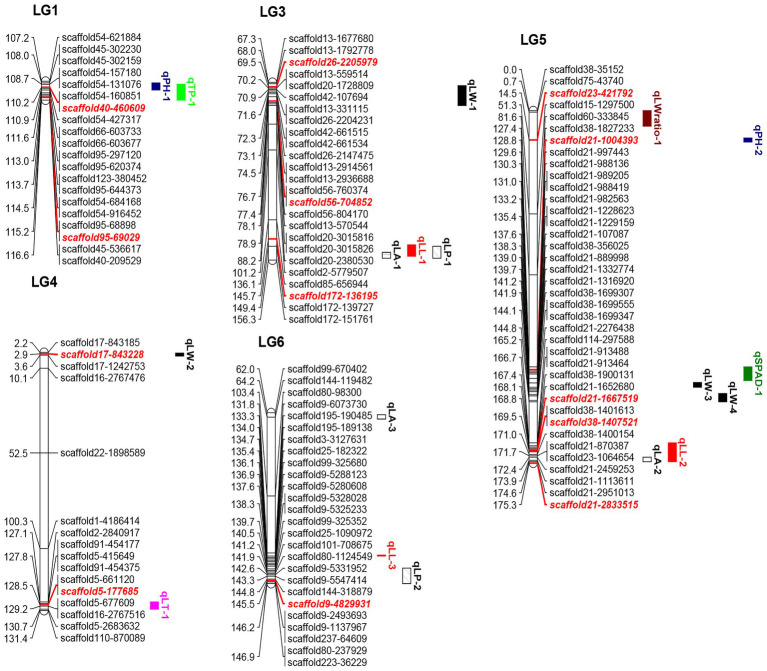
Quantitative trait loci locations on the linkage map. The red and italicized loci correspond to SNPs validated in KASP experiments. LL, leaf length; LW, leaf width; LA, leaf area; LW ratio, LL/LW; LP, leaf perimeter; TP, trunk perimeter; LT, leaf thickness; SPAD, chlorophyll content; and PH, plant height.

To validate the identified SNPs, 50 individuals were selected with the KASP technology. Of the 15 selected SNP pairs, 13 were genotyped successfully in these 50 individuals (Supplementary Table 2; Figure 1), with a 94.92% success rate (617/650; Supplementary Table 3), confirming the accuracy of detected SNPs.

### Linkage Group Construction

**Figure 2:**
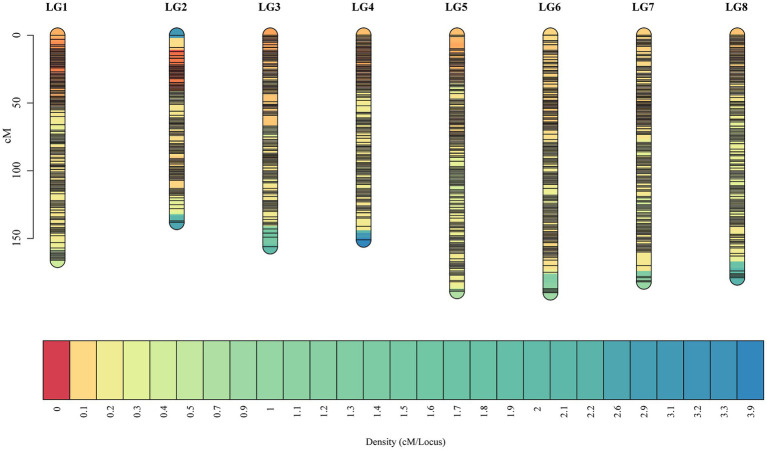
The high-density F_1_ population-based integrated genetic map. The *x*-axis corresponds to the linkage group, and the *y*-axis corresponds to the location, with marker density increasing gradually from red to blue.

Selected markers were first used to construct male and female maps. The female map contained 22,218 polymorphic markers in 8LGs, with a 1,854.93 cM total distance and a 0.08 cM average marker interval distance, with LGs, ranging from a 173.39‐ to 304.41-cM long and with average distance, ranging from 0.04 (LG2) to 0.16 (LG8) cM. The male map contained 20,261 markers grouped into 8 LGs, with a 1,468.04-cM total distance and an average 0.07-cM marker interval distance, with LGs ranging from 138.33‐ to 252.31-cM long and with average marker distances ranging from 0.03 (LG2) to 0.17 (LG8) cM (Supplementary Table 4). These two individual maps were then merged to generate an integrated map, containing 31,431 markers, with polymorphic markers having been classified into four segregation patterns, including “nn × np” (72), “lm × ll” (12,764), “ab × cc” (18,526), and “cc × ab” (69). The resultant integrated map spanned 1,351.85 cM at an average distance of 0.04 cM. LGs in this map ranged from 138.33 cM (LG2, average distance of 0.02 cM) to 189.66 cM (LG6, average distance of 0.06 cM) in length, and the number of markers per LG ranged from 2,126 (LG8, average distance of 0.08 cM) to 6,390 (LG1, average distance of 0.03 cM; Table 1; Figure 2).

**Table 1:** Genetic and physical map details.

Linkage group	No. of marker	Genetic distance (cM)	Average distance (cM)	Physical distance (Mb)
LG1	6,390	166.39	0.03	41.97
LG2	6,304	138.33	0.02	26.41
LG3	4,080	156.25	0.04	33.89
LG4	3,599	151.38	0.04	30.40
LG5	3,236	188.67	0.06	37.94
LG6	3,126	189.66	0.06	35.88
LG7	2,570	182.45	0.07	31.94
LG8	2,126	178.70	0.08	36.93
Total	31,431	1,351.85	0.04	275.37

### Physical Map Assembly and Comparative Analysis

**Figure 3:**
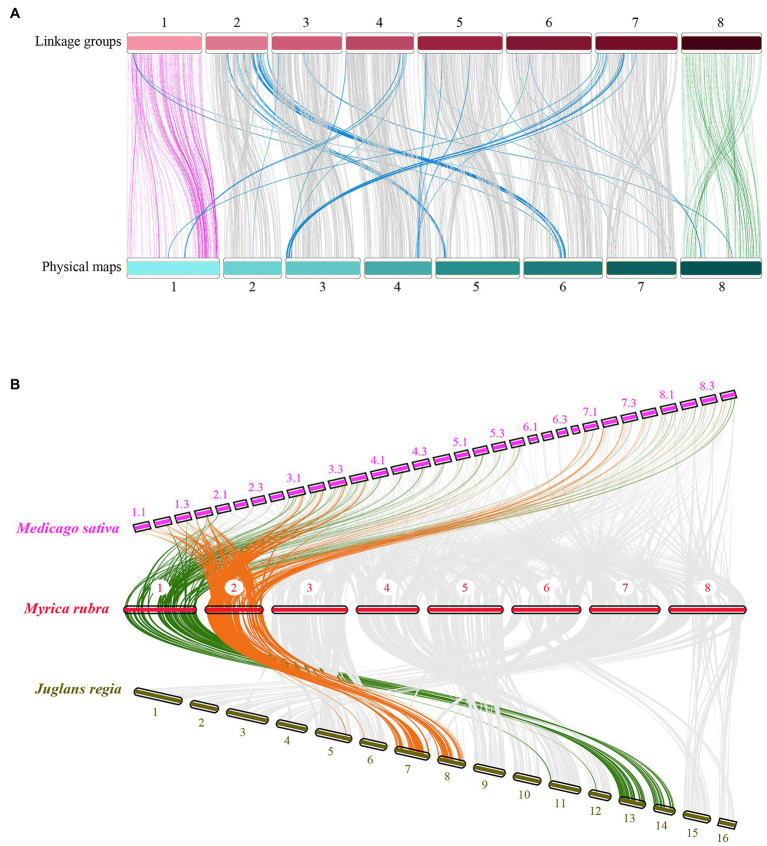
Comparative analysis of the *Myrica rubra* genome. **(A)** The collinearity of chromosomes between the genetic map and the physical map, 1–8 represent the eight chromosomes. **(B)** Syntenies among *M. rubra* (pseudochromosomes 1–8), *Jugians regia* (chromosomes 1–15), and *Medicago sativa* (chromosomes 1–8). In *M. sativa*, each chromosome was divided into four parts (e.g., 1.1, 1.2, 1.3, and 1.4).

Based on the prior scaffold genome (Ren et al., 2019) and the collinear sites in each LG, all 31, 431 SNPs in this map were anchored in the reference genome to assess the collinearity of the genetic LGs, revealing a high degree of collinearity between these LGs and the corresponding physical maps (Figure 3A). However, there were certain inconsistent regions on several chromosomes, including an inverted scaffold on chromosomes 5, 6, 7, and 8. The Chinese bayberry genome was ultimately assembled into eight pseudo-chromosomes and was 275.37 Mb in length, comprising 94.98% of the total assembly (289.92 Mb; Table 2; NCBI Sample Project No. SUB8537347). Chromosome sizes ranged from 26.41 to 41.97 Mb.

**Table 2:** Biqizhong, 2012LXRM, and F_1_ population phenotypic information.

Traits	Biqizhong	2012LXRM	F_1_ Population					
			Min	Max	Mean	SD	CV (%)	Skew
LL (cm)	10.83	15.92	6.00	17.37	10.40	1.94	18.61	0.68
LW (cm)	3.10	4.63	1.60	5.03	3.56	0.47	13.20	0.08
LW ratio	3.49	3.44	2.81	5.13	3.77	0.43	11.37	0.54
LA (cm^2^)	39.11	69.78	17.61	73.35	36.51	9.52	26.08	0.83
LP (cm)	23.82	33.22	13.25	36.93	22.52	4.09	18.15	0.53
LT (mm)	0.26	0.36	0.26	0.51	0.36	0.06	15.57	0.87
SPAD	47.14	44.75	22.07	51.60	39.41	5.75	14.59	−0.40
PH (cm)	250.00	300.00	94.00	261.00	174.81	29.17	16.69	−0.29
TP (cm)	65.00	68.00	6.80	17.00	12.37	1.84	14.85	−0.44

LL, leaf length; LW, leaf width; LA, leaf area; LW ratio, LL/LW; LP, leaf perimeter; TP, trunk perimeter; LT, leaf thickness; SPAD, chlorophyll content; and PH, plant height.

We then calculated comparative syntenic relationships in *M. rubra*, *Jugians regia*, and *Medicago sativa*. In total, 302 (*M. rubra* vs. *Jugians regia*) and 948 (*M. rubra* vs. *Medicago sativa*) orthologous gene pair blocks were detected and used as a means of visualizing orthologous chromosome-to-chromosome relationships (Figure 3B). The complex syntenic patterns observed were indicative of high levels of chromosomal rearrangement between *M. rubra* and *Jugians regia*, while each bayberry chromosome was largely consistent with the four birch chromosomes of *Medicago sativa*.

### Leaf and Growth Trait Performance

Table 2 compiles seven leaf‐ and two plant-growth-related traits. The LL, LW, LA, and LP of 2012LXRM were larger and wider than those of Biqizhong. A wide range of variation in the nine analyzed traits was observed in the F_1_ population. The coefficient of variation (CV) values of LA (26.08%) were higher than those for other traits, whereas the CV for the LW ratio was the lowest (11.37%). There were significant differences in leaf traits (Supplementary Figure 2). As shown in Table 3, positive correlations (*p* = 0.01) between LL and LW, LL, and LW ratio, LL and LA, LL and LP, LW and LA, LW and LP, LW and LT, LW and TP, LW ratio and LA, LW ratio and LP, LA and LP, LT and PH, LT and TP, SPAD and PH, SPAD and TP, and PH and TP were found to be statistically significant. In addition, significant negative correlations (*p* = 0.01) were detected between LL and SPAD, LW ratio and LT, LW ratio and SPAD, LW ratio and PH, LW ratio and TP, and LP and SPAD.

**Table 3:** Correlations among nine traits in the F_1_ population.

Traits	LL	LW	LW ratio	LA	LP	LT	SPAD	PH	TP
LL	1								
LW	0.79^∗∗^	1							
LW ratio	0.42^∗∗^	−0.22^∗^	1						
LA	0.96^∗∗^	0.86^∗∗^	0.26^∗∗^	1					
LP	0.98^∗∗^	0.83^∗∗^	0.32^∗∗^	0.96^∗∗^	1				
LT	0.03	0.28^∗∗^	−0.34^∗∗^	0.05	0.09	1			
SPAD	−0.23^∗∗^	−0.10	−0.24^∗∗^	−0.20^∗^	−0.24^∗∗^	0.19^∗^	1		
PH	−0.01	0.21^∗^	−0.33^∗∗^	0.04	0.01	0.26^∗∗^	0.52^∗∗^	1	
TP	0.06	0.25^∗∗^	−0.31^∗∗^	0.11	0.08	0.23^∗∗^	0.47^∗∗^	0.70^∗∗^	1

LL, leaf length; LW, leaf width; LA, leaf area; LW ratio, LL/LW; LP, leaf perimeter; TP, trunk perimeter; LT, leaf thickness; SPAD, chlorophyll content; and PH, plant height.

∗ Stands for the significant level *p* = 0.05.

∗∗ Stands for the significant level *p* = 0.01.

### Quantitative Trait Loci Analysis

The positions and percentages of the phenotypic variance explained (PVE) by QTL markers are shown in Table 4 and Figure 1. In total, 18 QTLs were identified and were found to explain 7.02–19.34% of trait variance, with an average PVE of 9.07% of the nine evaluated traits.

**Table 4:** Quantitative trait loci information.

Name of QTLs	Linkage group	Marker	Position (cM)	Range (cM)	LOD	Additive	Dominant	PVE (%)
qLA-1	3	scaffold172-139,727	155.38	152.38–155.38	2.70	4.69	−5.76	8.19
qLA-2	5	scaffold21-1,113,611	173.87	172.42–174.59	2.65	1.85	2.88	8.57
qLA-3	6	scaffold144-119,482	64.21	63.02–65.21	2.60	4.36	−5.17	7.65
qLL-1	3	scaffold172-139,727	150.38	148.68–154.38	2.58	0.81	−1.12	7.82
qLL-2	5	scaffold21-2,459,253	173.42	165.21–174.59	3.06	0.39	0.62	9.42
qLL-3	6	scaffold195-190,485	133.27	132.82–133.27	2.64	0.22	−1.09	7.78
qLP-1	3	scaffold172-139,727	152.38	149.38–155.38	2.41	1.59	−2.33	7.51
qLP-2	6	scaffold223-36,229	146.96	139.30–146.96	2.85	−0.33	−1.52	8.58
qLT-1	4	scaffold5-177,685	128.53	127.09–130.69	2.64	−0.11	−0.03	7.63
qLW-1	3	scaffold26-2,205,979	69.47	69.02–78.87	3.23	0.23	−0.24	8.87
qLW-2	4	scaffold17-843,228	2.90	2.50–4.00	2.60	0.19	−0.24	7.02
qLW-3	5	scaffold21-107,087	137.58	135.20–137.58	2.92	−0.73	0.18	8.83
qLW-4	5	scaffold38-1,699,307	143.90	140.73–144.81	3.03	−0.10	0.28	9.54
qLW ratio-1	5	scaffold75-43,740	0.72	0.01–7.73	3.24	0.77	−0.27	10.75
qPH-1	1	scaffold40-460,609	110.14	107.97–111.58	3.00	5.18	−17.68	9.20
qPH-2	5	scaffold23-421,792	14.48	13.58–15.58	2.58	−17.31	13.80	8.01
qSPAD-1	5	scaffold21-997,443	129.57	127.40–134.2	2.97	0.79	2.04	8.47
qTP-1	1	scaffold40-460,609	110.14	108.69–116.63	4.28	1.53	−1.28	19.34

LL, leaf length; LW, leaf width; LA, leaf area; LW ratio, LL/LW; LP, leaf perimeter; TP, trunk perimeter; LT, leaf thickness; SPAD, chlorophyll content; and PH, plant height.

There were three LA-related QTLs detected in LG3 (152.38–155.38 cM), LG5 (172.42–174.59 cM), and LG6 (63.02–65.21 cM), with LODs ranging from 2.60 to 2.70 and PVEs from 7.65 to 8.57%. Three LL-related QTLs were located in LG3 (148.68–154.38 cM), LG5 (165.21–174.59 cM), and LG6 (132.82–133.27 cM), with LOD values of 2.58, 3.06, and 2.64, explaining 7.82, 9.42, and 7.78% of the observed genotypic variation, respectively. In addition, two LP-related QTLs were identified in LG3 (149.38–155.38 cM) and LG6 (139.3–146.96 cM), with LOD values of 2.41 (threshold value = 2.35) and 2.85, respectively, explaining 7.51 and 8.58% of the observed genotypic variation. Additionally, four LW-related QTLs were detected in LG3 (69.02–78.87 cM), LG4 (2.5–4 cM), and LG5 (135.2–137.58 and 140.73–144.81 cM), with LODs ranging from 2.60 to 3.23, and with PVEs from 7.02 to 9.54%. Two PH-related QTLs were detected in LG1 (107.97–111.58 cM) and LG5 (13.58–15.58 cM), with LOD values of 3.00 and 2.58, explaining 9.20 and 7.63% of PVE. One QTL each was associated with LT, LW ratio, SPAD, and TP, which were mapped to LG4 (127.09–130.69 cM), LG5 (0.01–7.73 cM), LG5 (127.4–134.2 cM), and LG1 (108.69–116.63 cM), explaining 7.63, 10.75, 8.47, and 19.34% of PVE, respectively.

### Quantitative Trait Loci Clusters, Candidate Gene Prediction, and Expression Analyses

Quantitative trait loci clusters are regions of the chromosome containing multiple loci associated with a range of traits (Kochevenko et al., 2018; Guan et al., 2020). Herein, we detected two QTL clusters on two LGs (Table 5). The LG3 cluster contained three QTLs, and its approximate position was 148.68–155.38 cM. The leaf trait-related *MrChr3G1120*, *MrChr3G1121*, and *MrChr3G1125* candidate genes were within this region. The LG5 cluster contained three QTLs with an approximate position of 129.57–143.90 cM. The *MrChr5G3156*, *MrChr5G3291*, *MrChr5G3292*, *MrChr5G3258*, *MrChr 5G3089*, and *MrChr5G3034* candidate genes were found within this region and were associated with cytochrome P450 and chlorophyll a/b-binding protein functionality.

**Table 5:** Quantitative trait loci clusters and candidate gene functional predictions.

QTL clusters	Range (cM)	QTL name	Candidate genes	Function annotation
LG3-cluster	148.68–155.38	qLL-1	*MrChr3G1120*	Hypothetical protein PRUPE_ppa004259mg
		qLA-1	*MrChr3G1121*	Bidirectional sugar transporter SWEET3
		qLP-1	*MrChr3G1125*	ATP-dependent zinc metalloprotease FTSH 11, chloroplastic/mitochondrial
LG5-cluster	129.57–143.90	qLW-3	*MrChr5G3156*	Chlorophyll a/b-binding protein
		qLW-4	*MrChr5G3292*	Ocytochrome P450 76A1-like
		qSPAD-1	*MrChr5G3291*	Ocytochrome P450 76A1
			*MrChr5G3258*	Growth-regulating factor 3
			*MrChr5G3089*	Ovate family protein
			*MrChr5G3034*	Peptidyl-prolylcis-trans isomerase FKBP13, chloroplastic

LL, leaf length; LW, leaf width; LA, leaf area; LP, leaf perimeter; and SPAD, chlorophyll content.

**Figure 4:**
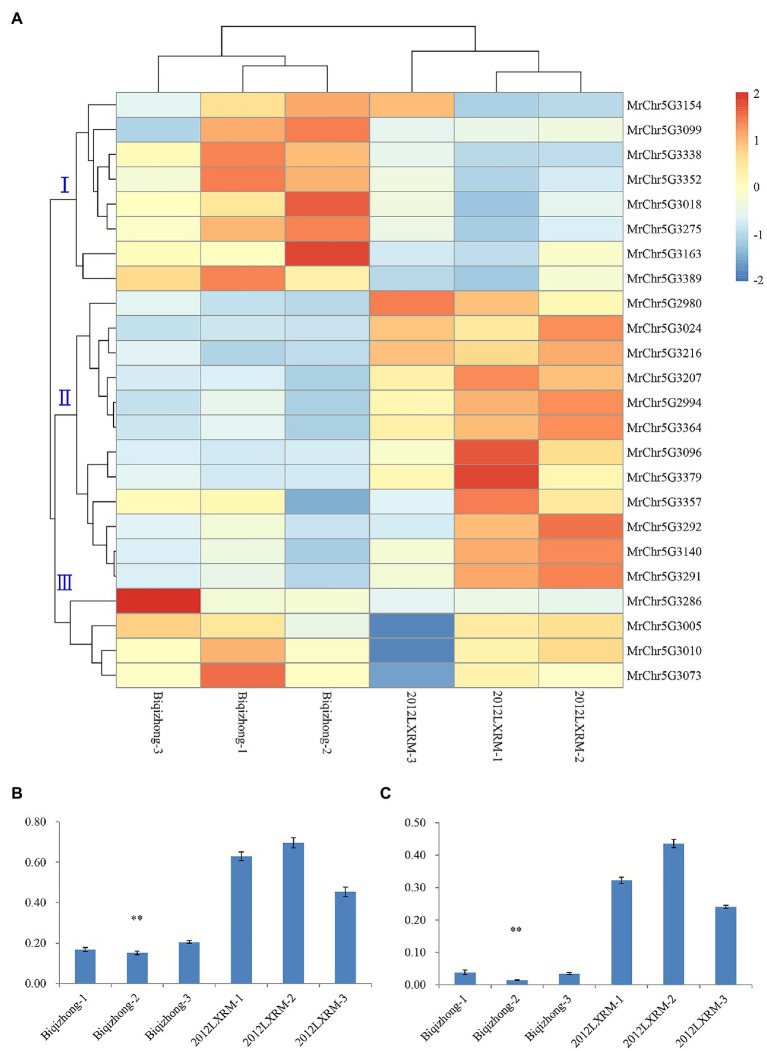
The expression of 24 differentially expressed genes **(A)**. Relative expression of the candidate genes MrChr5G3291 **(B)** and MrChr5G3292 **(C)** in the LG5-cluster region. **(A)** Groups I, II, and III are three groups clustered according to gene sequence; blue and red represent downregulation and upregulation of gene expression, respectively; Biqizhong 1–3 and 2012LXRM 1–3 represent three replicate parental leaves. ^∗∗^*p* = 0.01.

An RNA-seq approach was then used to conduct transcriptomic analyses of leaf-related genetic traits in bayberry plants. In total, these analyses yielded 39.62 Gb of clean data from six-leaf samples obtained by transcriptome analysis of six-leaf samples (Supplementary Table 5). In total, 732 and 741 genes were up and downregulated in Biqizhong leaves relative to 2012LXRM leaves, respectively. In total, 24 differentially expressed genes were identified within the LG5-cluster region, and these genes were separated into three groups (Supplementary Table 6; Figure 4A). Groups I and III were composed of eight and four genes that were downregulated in 2012LXRM and upregulated in Biqizhong, while group II contained 12 genes, including *MrChr5G3291* and *MrChr5G3292*, which were related to leaf traits, and which were upregulated in 2012LXRM and downregulated in Biqizhong.

RNA-seq results for 10 genes were further validated *via* qPCR using (Supplementary Table 7; Supplementary Figure 3). Importantly, qPCR-based analyses of the expression of the differentially expressed candidate genes *MrChr5G3291* and *MrChr5G3292* confirmed that they were significantly differentially expressed in Biqizhong and 2012LXRM leaf samples (Figures 4B,C). The CDS of *MrChr5G3291* and *MrChr5G3292* were consistent with members of the cytochrome P450 superfamily, which is among the largest families of enzymes in plants, and which are involved in a range of processes, including signaling, structural maintenance, and defense-compound production. In particular, P450 superfamily genes are involved in dioxobilin-type chlorophyll catabolite formation (Christ et al., 2013), and were present in the qSPAD-1 QTL in the LG5 cluster. Therefore, we speculate that *MrChr5G3291* and *MrChr5G3292* maybe leaf color-related genes in bayberry plants.

## Discussion

Genetic linkage maps are important as a tool for clarifying the genetic basis for key traits of interest and for developing additional DNA-based diagnostic tools or MAS-based breeding strategies. Herein, Illumina sequencing approaches were employed to facilitate the re-sequencing of 140 F_1_ Chinese bayberry progeny and their parents, yielding 902.11 Gb of raw data from which a high-density genetic map was successfully constructed. Relative to other previously published maps (Wang et al., 2020), our map is superior with respect to population size (140 vs. 95), sequencing method (Re-sequencing vs. RAD-sequencing), sequence data (902.11 vs. 247.64 Gb), and marker number (31,431 vs. 3,191; Supplementary Table 8). The number of markers in our map was roughly 10-fold higher than this prior map, and such increased density enables more accurate QTL detection and can better facilitate potential candidate genes associated with key plant traits. This map can serve as a high-quality reference to support future molecular breeding, genetics, and evolutionary studies of Chinese bayberry.

Genetic maps offer value as a means of anchoring scaffolds to pseudo-chromosomes for species that lack organized chromosome-level genetic information the genome of the species is not yet organized at the chromosome level (Tang et al., 2015). Using a genetic map, a bayberry cv. Zaojia reference genome was assembled. As compared with the previous bayberry cv. Y2012-145 draft genome (Jiao et al., 2012; Jia et al., 2019), this genome (Ren et al., 2019) exhibited high-quality sequence assembly with respect to sequencing depth (45.01× > 26×), assembly sequence coverage (95.25 > 79.16%) and N50 sequence length (68.65 kb > 295 bp; Supplementary Table 9). Collinearity in Minimap2 (Li, 2018) was also observed relative to the previous bayberry cv. Y2012-145 draft genome (Jiao et al., 2012) assembled based on a 3,075 SNP marker genetic map, with some inversions and sequence differences being evident between these two versions (Supplementary Figure 4). As these two genomes were assembled using genetic maps, sequence correction is not possible and so the accuracy of splicing cannot be readily established. Future studies should leverage the Hi-C scaffolding technology to prepare a high-quality Chinese bayberry reference genome.

For QTL mapping of the F_1_ population, we specifically focused on nine leaf‐ and growth-associated traits, leading to the detection of 18 QTLs, explaining approximately 9.07% of the variability in these traits. Of these, 15 QTLs were associated with leaf traits, while three were associated with growth traits. In addition, we detected two QTL clusters, each containing three QTLs. Such clusters are of particular relevance to breeders, as they can thereby focus their efforts on QTL regions associated with the greatest degree of phenotypic variance. In total, nine potentially relevant genes were located within these two cluster ranges. RNA-seq and qPCR-based validation efforts led to the identification of *MrChr5G3291* and *MrChr5G3292* as candidate leaf color-trait related genes. These genes are predicted to encode cytochrome P450, which is a key regulator of leaf development, internode elongation, leaf cuticular wax synthesis, and dioxobilin-type chlorophyll catabolite formation (Miyoshi et al., 2004; Christ et al., 2013; Jáquez-Gutiérrez et al., 2019; Zhang et al., 2020). These two candidate genes thus represent ideal targets for future cloning and functional verification studies. Other genes in this cluster include *MrChr5G3156*, which is a predicted chlorophyll a/b-binding protein expressed in green tissues and linked to photosynthesis in *Jatropha curcas* L., Euphorbiaceae (Zhao et al., 2020). *MrChr5G3258* was also in this cluster and was annotated as growth-regulating factor 3, participating in essential processes such as root and leaf development, seed and flower formation, inflorescence, and hormone network regulation in plants (Chen et al., 2020). *MrChr5G3089* was annotated as an OVATE family protein, the overexpression of which resulted in altered plant morphology, leaf chlorophyll accumulation, and retarded leaf senescence in tomatoes (Zhou et al., 2019). The specific roles of these genes in *M. rubra* will be a focus of future research efforts.

In summary, we herein developed a high-density genetic map, containing 31,431 SNP markers based on there-sequencing of 140 F_1_ Chinese bayberry plants. We then utilized this map to prepare a draft genome assembled into eight pseudo-chromosomes, and we identified 18 QTLs associated with key leaf and growth traits, as well as two relevant QTL clusters and two candidate genes. This study is the first QTL mapping analysis of *M. rubra* to our knowledge. Our high-density map and reference genome tools may prove invaluable for future mapping efforts, evaluating fruit quality, sex determination, and stress resistance traits, and will assist the identification of functional markers. The candidate genes within the identified QTLs in the present study may be key regulators of leaf size, color, and bayberry development and will be important topics of future research. Overall, our study provides a robust foundation for the MAS-based breeding of Chinese bayberry.

## Data Availability Statement

The data presented in the study are deposited in SRA (http://www.ncbi.nlm.nih.gov/bioproject/733585), and accession number is PRJNA733585.

## Author Contributions

SZ and XQ conceived and designed the experiments. SZ performed the experiments. SZ and ZY analyzed the data and wrote the manuscript. ZW, YZ, HR, SL, and XZ participated in the experiments and analysis. SZ, XQ, and ZY edited the manuscript. All authors contributed to the article and approved the submitted version.

### Conflict of Interest

The authors declare that the research was conducted in the absence of any commercial or financial relationships that could be construed as a potential conflict of interest.

## Footnotes

^1^www.cerealsdb.uk.net

^2^https://sourceforge.net/projects/lep-map3/
