# Supplementary material for: Construction of a High-Density Genetic Map and Identification of Leaf Trait-Related QTLs in Chinese Bayberry (Myrica rubra)
Source: Front Plant Sci. 2021 Jun 14;12:675855. doi: 10.3389/fpls.2021.675855 (PMC8238045; doi:10.3389/fpls.2021.675855)
Supplement: Supplementary file 1 [file Data_Sheet_1.docx]

**Supplementary Figures**

Biqizhong

2012LXRM


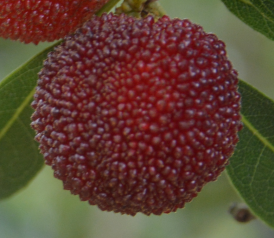

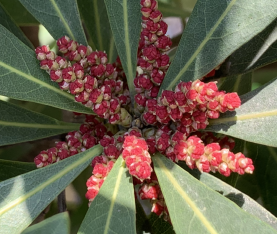


F_1_

Seeds of F_1_ progeny were sown


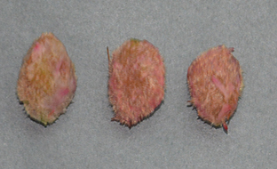

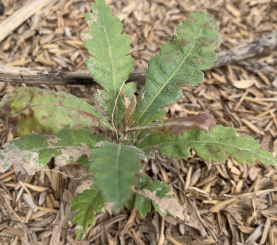


F_1_ individual were transplanted


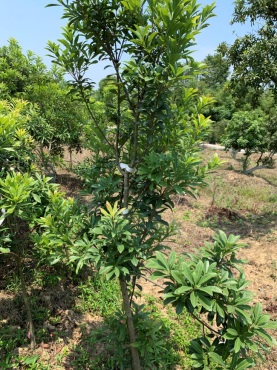


Leaf samples were collected

Leaf and growth trait were measured

**Supplementary Figure 1. The flow chart of F_1_ population.** In 2012, pollen was collected from these two parental plants for hybridization, and F_1_ progeny seeds were sown. In 2015, all F_1_ individuals were transplanted to an experimental field. In 2019, young healthy leaf samples from 140 F_1_ individuals and from both parental plants were then collected. In 2020, F_1_ plant leaf parameters and growth traits were assessed, with analyses being specifically conducted using second-year leaves on the first-order branches of the main stem.


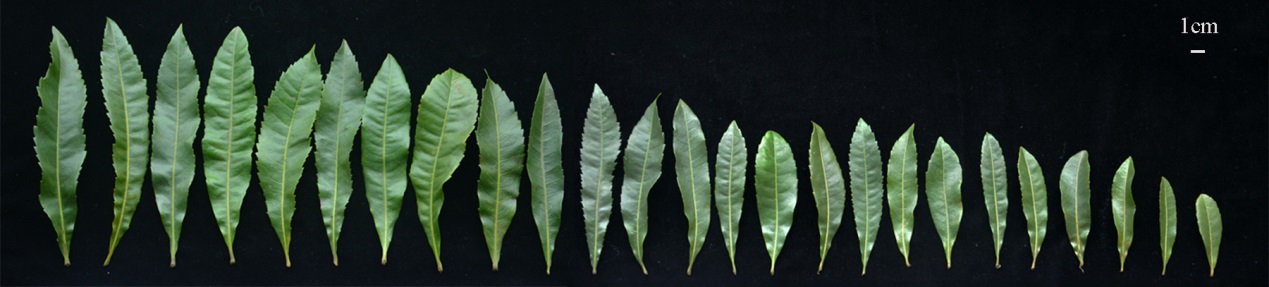


**Supplementary Figure 2. The leaf of some F_1_ individuals at the same position.** The LL, LW, LWratio, LA, LP, LT and SPAD were significant differences.


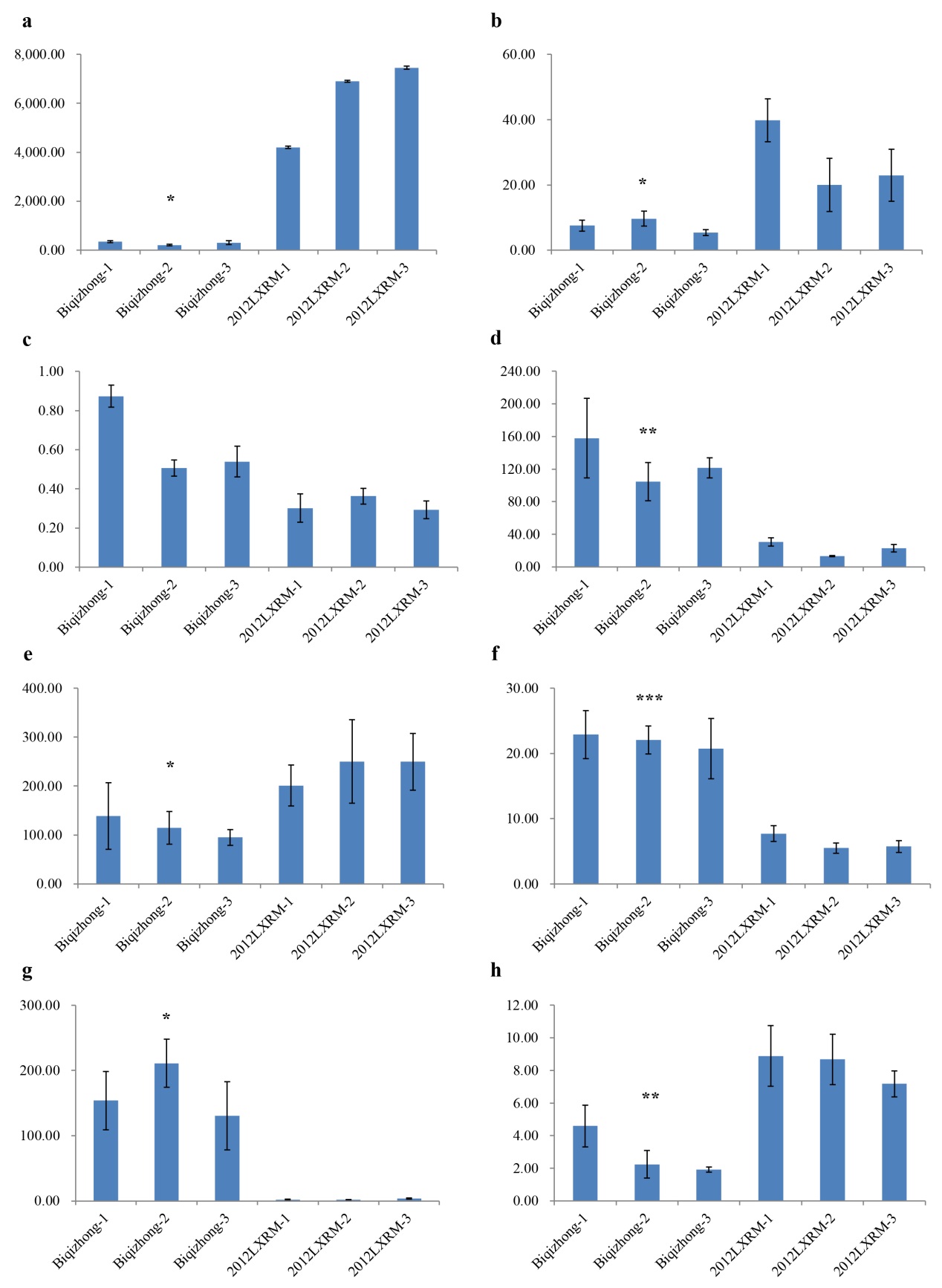


**Supplementary Figure 3. Relative expression of differentially expressed genes in LG5-cluster range.** (a-h) The relative expression of *MrChr5G2994*, *MrChr5G3024*, *MrChr5G3073*, *MrChr5G3163*, *MrChr5G3207*, *MrChr5G3275*, *MrChr5G3352*, and *MrChr5G3379* in Biqizhong and 2012LXRM, respectively. *: stands for the significant level p = 0.05; **: stands for the significant level p = 0.01.


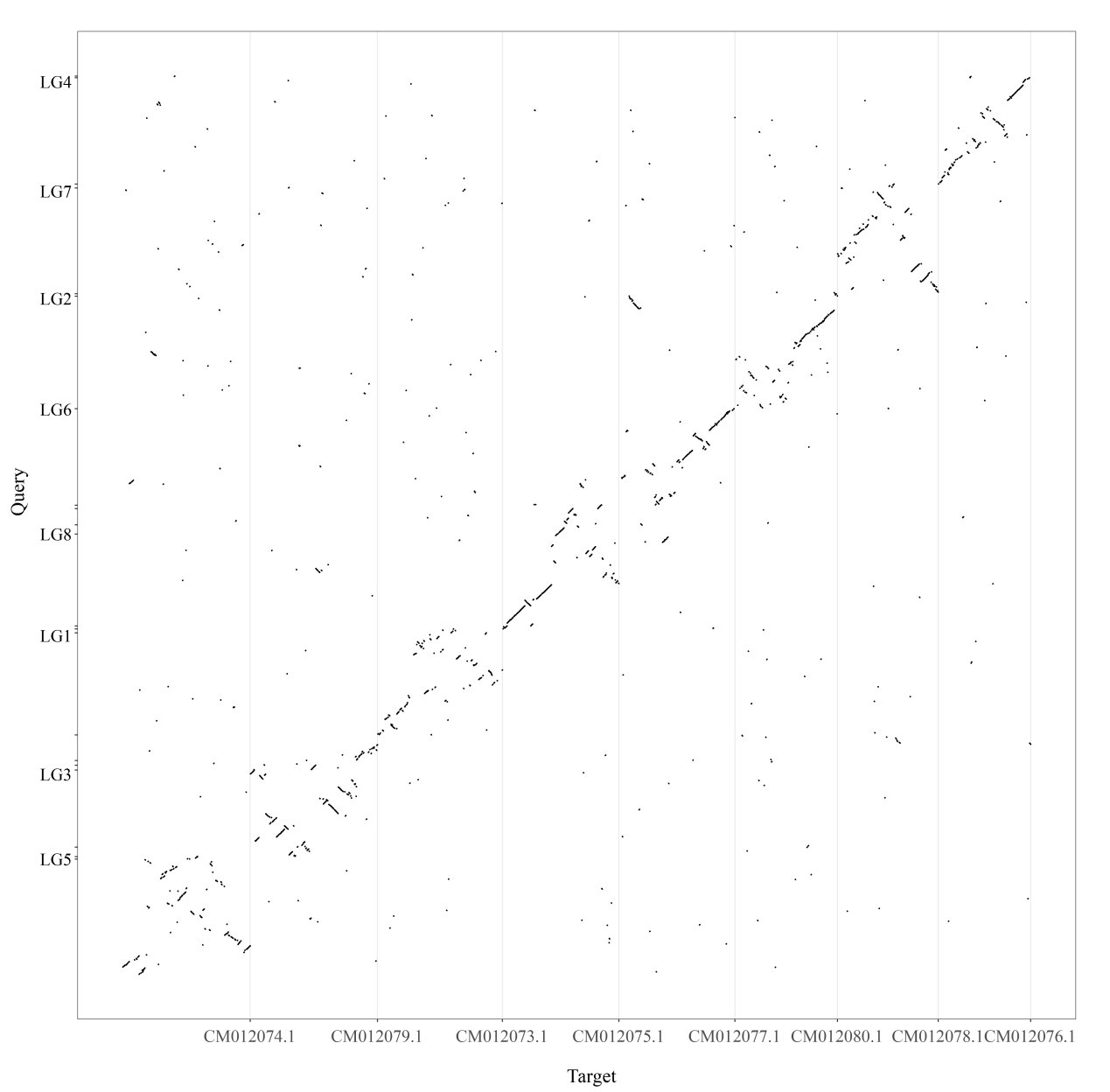


**Supplementary Figure 4. Comparison of chromosome sequences between Zaojia and Y2012-145.** The ordinate is the chromosome number of this study, and the abscissa is chromosome of Y2012-145.

**Supplementary Tables**

**Supplementary Table 1. Summary of re-sequence data**

|  | Biqizhong | 2012LXRM | F_1_ Population | ***Average***/Total |
| --- | --- | --- | --- | --- |
| Clean base (bp) | 8,634,550,800 | 5,770,470,300 | 6,340,731,936 | 902,107,492,200 |
| No. of clean reads | 57,563,672 | 38,469,802 | 42,272,702 | **-** |
| GC content (%) | 37.83 | 38.01 | 38.21 | ***38.2*** |
| Q30 (%) | 93.12 | 92.69 | 92.24 | ***92.25*** |
| Average depth | 22.09 | 17 | 16.92 | ***16.95*** |
| No. of SNPs | 2,061,013 | 2,094,875 | 2,162,652 | 2,242,353 |

**Supplementary Table 2. Information of 13 KASP-SNP markers**

| Primer name | Primer_AlleleFAM | Primer_AlleleHEX | Primer_Common | AlleleFAM | AlleleHEX |
| --- | --- | --- | --- | --- | --- |
| scaffold40-460609 | GAAGGTGACCAAGTTCATGCTCTTCCTGTCAAGCTTTTAAAAC | GAAGGTCGGAGTCAACGGATTCTTCCTGTCAAGCTTTTAAAAT | GATAGTTATAATTTTATATTCAAAGTAAGA | C | T |
| scaffold95-69029 | GAAGGTGACCAAGTTCATGCTTGGAGGATACGAACGCCGA | GAAGGTCGGAGTCAACGGATTTGGAGGATACGAACGCCGG | AGTATCTTCAAAAACTGAAACTAGAA | A | G |
| scaffold26-2205979 | GAAGGTGACCAAGTTCATGCTAAGAGAGTTAGGAGGGTTTGGGCCG | GAAGGTCGGAGTCAACGGATTAAGAGAGTTAGGAGGGTTTGGGCCA | TGGCTAAATCTTTTTTTTTCTCTCTC | G | A |
| scaffold56-704852 | GAAGGTGACCAAGTTCATGCTATCTACCGACTCCTAGACCTCTA | GAAGGTCGGAGTCAACGGATTATCTACCGACTCCTAGACCTCTG | TGGCAACAACATGTCTATGGAGCATA | A | G |
| scaffold172-136195 | GAAGGTGACCAAGTTCATGCTCTGCGATTTCTGACAGATGTT | GAAGGTCGGAGTCAACGGATTCTGCGATTTCTGACAGATGTA | GAGCAATAAAACCAGTATGGAACTGAAAAA | T | A |
| scaffold17-843228 | GAAGGTGACCAAGTTCATGCTGGGTTTAGGATATTCTCCAATT | GAAGGTCGGAGTCAACGGATTGGGTTTAGGATATTCTCCAATG | TCCATCTCTCGAAATTGAACCA | T | G |
| scaffold5-177685 | GAAGGTGACCAAGTTCATGCTGTGGATTCAATGTGTTAAAGGTGAT | GAAGGTCGGAGTCAACGGATTGTGGATTCAATGTGTTAAAGGTGAC | GCCCTCTAACTACCAATATGATTTTA | T | C |
| scaffold23-421792 | GAAGGTGACCAAGTTCATGCTAACAGCTCAGGTGTCCGCCTTACA | GAAGGTCGGAGTCAACGGATTAACAGCTCAGGTGTCCGCCTTACC | TATGATGCGTGATCAGCAACCG | A | C |
| scaffold21-1004393 | GAAGGTGACCAAGTTCATGCTTGCTTGAAGGGCGAATGAC | GAAGGTCGGAGTCAACGGATTTGCTTGAAGGGCGAATGAA | CCTCAGATCTTCCCTATTGTCACTTGGTTC | C | A |
| scaffold21-1667519 | GAAGGTGACCAAGTTCATGCTGTAGGTTGTGCAAGTTTCG | GAAGGTCGGAGTCAACGGATTGTAGGTTGTGCAAGTTTCA | TTTGTAACATTAAATTGGGATGG | G | A |
| scaffold38-1407521 | GAAGGTGACCAAGTTCATGCTTAGCCCGGGATTCAGCATACAACTC | GAAGGTCGGAGTCAACGGATTTAGCCCGGGATTCAGCATACAACTG | AGAACCCTACATTAGGTAAGATTCCA | C | G |
| scaffold21-2833515 | GAAGGTGACCAAGTTCATGCTTCAAGGGTTGGTGATGTGGGAGTA | GAAGGTCGGAGTCAACGGATTTCAAGGGTTGGTGATGTGGGAGTG | GACTAAATAGACCTGATAAAGCAGCTTGCA | A | G |
| scaffold9-4829931 | GAAGGTGACCAAGTTCATGCTGCCAGTTAGAGACAAAATTTG | GAAGGTCGGAGTCAACGGATTGCCAGTTAGAGACAAAATTTA | TGCTGTTGAAACCATCAGTGGTAAAG | G | A |

**Supplementary Table 3. Comparison of KASP-SNP and re-sequencing SNP in 50 F_1_ individuals**

| Sample Name | scaffold5-177685 | scaffold9-4829931 | scaffold17-843228 | scaffold21-1004393 | scaffold21-1667519 | scaffold21-2833515 | scaffold23-421792 | scaffold26-2205979 | scaffold38-1407521 | scaffold40-460609 | scaffold56-704852 | scaffold95-69029 | scaffold172-136195 |
| --- | --- | --- | --- | --- | --- | --- | --- | --- | --- | --- | --- | --- | --- |
| Female | T & C | G & A | T & G | C & A | G & A | A & G | A & C | G & A | C & G | C & T | A & G | A & G | T & A |
| Male | C | A | T | A | A | G | C | A | G | T | G | G | A |
| 1 | T | G | T & G | C & A | G | A & G | A & C | G | C & G | C & T | A | A & G | T |
| 2 | T & C | G & A | T | C & A | G & A | A | A & C | G | C | C & T | A | A & G | T |
| 3 | C | A | T & G | C | G & A | G | C | G | G | C & T | A & G | A & G | T |
| 4 | **T & C(CC)** | A | G | C | G & A | **A & G(GG)** | **C(AC)** | **A(GA)** | C & G | **C(TC)** | A & G | A & G | **A(TA)** |
| 5 | T & C | G & A | T & G | C | G & A | G | C | G & A | G | C | A & G | A & G | A |
| 7 | C | G & A | G | C & A | G & A | G | A & C | G & A | G | C | A & G | A & G | A |
| 8 | T | G | T & G | C | G | A & G | C | A | C & G | C | G | A | T & A |
| 9 | T & C | G & A | T | C & A | G & A | A | A & C | G & A | C | T | A & G | G | T & A |
| 10 | C | G & A | **T(GT)** | C | G | **A(AG)** | C | A | **G(GC)** | C & T | **A(AG)** | A | T & A |
| 13 | C | A | T & G | C | G & A | G | C | G & A | G | T | G | A | T & A |
| 14 | T | **G(GA)** | T & G | C | G | A & G | **A(AC)** | **A(GG)** | C & G | C | A | A | T |
| 15 | T | A | T & G | C & A | A | A & G | C | G & A | C & G | C & T | A & G | A & G | T |
| 17 | T & C | G & A | G | C | G & A | G | A & C | A | C & G | C | G | A & G | T & A |
| 18 | C | G | T & G | C | G & A | **A(AG)** | **C(AC)** | G | C & G | T | A | A & G | T & A |
| 19 | T | G & A | T & G | C & A | G | A & G | A & C | G & A | C & G | T | A & G | G | T & A |
| 20 | T | G & A | T & G | C | G | A & G | A & C | G | C & G | T | A | G | T |
| 21 | T | G & A | T & G | C & A | A | A | A & C | G | C & G | C | A & G | A | T & A |
| 22 | C | G & A | T | C & A | A | A | A | G | C | C & T | A | A | T |
| 25 | T | A | T & G | C & A | A | A & G | C | G & A | C | C | A & G | A & G | T & A |
| 26 | T | G & A | T | C | G & A | G | A & C | G | C & G | C & T | A | A | T |
| 27 | T | **G(GA)** | T & G | C & A | G & A | **A & G(AA)** | **A(AC)** | G | C | T | A | A & G | A |
| 28 | T | G | T & G | C & A | G & A | A | A & C | G & A | C | C & T | A & G | A & G | T & A |
| 29 | T & C | G & A | T | C | G & A | A & G | A & C | G & A | C & G | C | A & G | A | A |
| 30 | T & C | A | T & G | A | A | A & G | A | G & A | C & G | C & T | A & G | A | T |
| 32 | T & C | G & A | T | A | A | A | A & C | G & A | C & G | C & T | A & G | A & G | A |
| 33 | T & C | A | T & G | **A(CA)** | **G & A(AA)** | A & G | A | G & A | C & G | **C(TC)** | **A(AG)** | A & G | T |
| 34 | C | G | T | C | G | G | A & C | G & A | G | C & T | A & G | A & G | T & A |
| 36 | T & C | A | T | C & A | G & A | G | C | G & A | G | C | A & G | A & G | T & A |
| 38 | C | G & A | G | C & A | G & A | A & G | C | G | C & G | T | A | A & G | T & A |
| 40 | T | G & A | T | C & A | G | A & G | A | A | C & G | C | G | A | T & A |
| 41 | T & C | G & A | T & G | C | G & A | G | A & C | G & A | C & G | C | A & G | A & G | T & A |
| 42 | C | A | T | C & A | G | G | A & C | G & A | C & G | C & T | A & G | A & G | A |
| 44 | T | G & A | T & G | C & A | G | A & G | C | G & A | C & G | C & T | A & G | A & G | T & A |
| 45 | T & C | G & A | T & G | A | A | A & G | A | G & A | C & G | T | G | A & G | A |
| 46 | C | G & A | T & G | A | A | A & G | A & C | G & A | C & G | C & T | G | A & G | T & A |
| 47 | C | G & A | G | C | G | A & G | A & C | G | G | C & T | A & G | A | A |
| 49 | C | G | T & G | A | A | A & G | **A(AC)** | G & A | **C(GC)** | C & T | A & G | A & G | **A(TA)** |
| 56 | C | A | T & G | C & A | G & A | G | A & C | A | G | C & T | G | A & G | T & A |
| 57 | T | G & A | T & G | A | G & A | A | A | A | C | T | G | G | A |
| 66 | T | G & A | T & G | A | G & A | A & G | A & C | G & A | C | C & T | G | A & G | T & A |
| 73 | T & C | G & A | T & G | C & A | G & A | A & G | A & C | A | C & G | C & T | G | A & G | T & A |
| 80 | T & C | G & A | G | A | A | A | A & C | G & A | C & G | C & T | A & G | G | T & A |
| 84 | **T & C(TT)** | A | **T(GT)** | C & A | G & A | A & G | A & C | G | C & G | C & T | A | A | T & A |
| 114 | T | G & A | G | A | A | A & G | A & C | G & A | C & G | C | A | A & G | T & A |
| 136 | C | G | G | **C & A(CC)** | G & A | **G(AG)** | A & C | G | C & G | **T(CC)** | A | A | T |
| 142 | T & C | G & A | T & G | A | A | A & G | A | G & A | C | C & T | A & G | A & G | T & A |
| 144 | C | G & A | T & G | A | A | A & G | A | G & A | C | C | A | A & G | T & A |
| 148 | T & C | G & A | T & G | C & A | G & A | G | A & C | A | C & G | T | G | G | A |
| 162 | T & C | G | T & G | C & A | G & A | A | A & C | G & A | C | C & T | A & G | A & G | T & A |
| 165 | T | G & A | T & G | A | **G & A(AA)** | A | A | G | C | C & T | **A(AG)** | A & G | **T(TA)** |

**Note:** The red font sites are the locus of KASP verification failure, the re-sequencing SNPs in brackets.

**Supplementary Table 4. The description of female and male genetic map**

| Linkage group | No. of marker | Genetic distance (cM) | Average distance (cM) |
| --- | --- | --- | --- |
| ***Female*** |  |  |  |
| LG1 | 5,339 | 288.82 | 0.05 |
| LG2 | 4,494 | 173.39 | 0.04 |
| LG3 | 2,875 | 232.23 | 0.08 |
| LG4 | 2,525 | 222.79 | 0.09 |
| LG5 | 2,301 | 304.41 | 0.13 |
| LG6 | 1,904 | 189.66 | 0.10 |
| LG7 | 1,669 | 264.93 | 0.16 |
| LG8 | 1,111 | 178.70 | 0.16 |
| Total | 22,218 | 1854.93 | 0.08 |
| ***Male*** |  |  |  |
| LG1 | 4,718 | 166.39 | 0.04 |
| LG2 | 4,246 | 138.33 | 0.03 |
| LG3 | 2,488 | 232.23 | 0.09 |
| LG4 | 2,103 | 156.25 | 0.07 |
| LG5 | 1,720 | 151.38 | 0.09 |
| LG6 | 2,036 | 188.67 | 0.09 |
| LG7 | 1,493 | 182.45 | 0.12 |
| LG8 | 1,457 | 252.31 | 0.17 |
| Total | 20,261 | 1468.04 | 0.07 |

**Supplementary Table 5. Summary of transcriptome sequencing of Biqizhong and 2012LXRM leaves**

| Samples | Clean reads | Clean bases (bp) | GC Content (%) | %≥Q30 (%) |
| --- | --- | --- | --- | --- |
| Biqizhong-1 | 21,403,050 | 6,390,283,060 | 47.35 | 92.68 |
| Biqizhong-2 | 20,776,437 | 6,193,566,414 | 47.38 | 93.55 |
| Biqizhong-3 | 27,227,360 | 8,115,985,288 | 47.30 | 93.79 |
| 2012LXRM-1 | 20,791,947 | 6,203,009,418 | 46.59 | 93.98 |
| 2012LXRM-2 | 20,525,594 | 6,116,028,864 | 47.39 | 94.08 |
| 2012LXRM-3 | 22,141,936 | 6,600,630,588 | 47.08 | 93.71 |

**Supplementary Table 6. The expression of 24 differentially expressed genes in LG5-cluster**

| Gene name | 2012LXRM-1 | 2012LXRM-2 | 2012LXRM-3 | Biqizhong-1 | Biqizhong-2 | Biqizhong-3 |
| --- | --- | --- | --- | --- | --- | --- |
| MrChr5G2980 | 27.31 | 18.15 | 24.99 | 4.03 | 4.05 | 9.59 |
| MrChr5G2994 | 0.26 | 0.43 | 0.50 | 0.00 | 0.00 | 0.00 |
| MrChr5G3005 | 0.33 | 0.10 | 0.42 | 4.28 | 4.24 | 4.58 |
| MrChr5G3010 | 0.00 | 0.00 | 0.00 | 4.77 | 4.29 | 3.54 |
| MrChr5G3018 | 5.69 | 23.78 | 18.69 | 65.10 | 91.11 | 40.76 |
| MrChr5G3024 | 4.88 | 9.78 | 6.07 | 1.92 | 2.93 | 1.92 |
| MrChr5G3073 | 1.01 | 0.00 | 0.00 | 8.11 | 5.35 | 4.42 |
| MrChr5G3096 | 15.23 | 9.00 | 3.43 | 1.95 | 3.30 | 2.90 |
| MrChr5G3099 | 3.07 | 3.26 | 3.40 | 12.49 | 13.43 | 5.93 |
| MrChr5G3140 | 0.00 | 0.00 | 0.00 | 0.51 | 0.37 | 0.67 |
| MrChr5G3154 | 6.08 | 6.90 | 8.74 | 17.68 | 18.39 | 12.05 |
| MrChr5G3163 | 0.93 | 2.08 | 2.24 | 7.03 | 11.22 | 6.72 |
| MrChr5G3207 | 3.97 | 2.74 | 2.21 | 1.51 | 1.39 | 1.98 |
| MrChr5G3216 | 90.73 | 124.23 | 69.30 | 24.51 | 29.76 | 42.74 |
| MrChr5G3275 | 3.39 | 6.73 | 6.90 | 28.06 | 29.61 | 15.32 |
| MrChr5G3286 | 6.35 | 2.67 | 1.03 | 19.55 | 21.08 | 112.35 |
| MrChr5G3291 | 0.00 | 0.00 | 0.00 | 0.45 | 0.71 | 0.80 |
| MrChr5G3292 | 0.02 | 0.26 | 0.11 | 1.83 | 2.19 | 1.85 |
| MrChr5G3338 | 3.42 | 4.08 | 7.17 | 42.74 | 34.04 | 20.78 |
| MrChr5G3352 | 2.41 | 16.24 | 20.08 | 135.91 | 103.37 | 39.71 |
| MrChr5G3357 | 3.22 | 1.89 | 1.84 | 5.97 | 4.22 | 5.65 |
| MrChr5G3364 | 0.87 | 1.49 | 1.04 | 0.29 | 0.20 | 0.52 |
| MrChr5G3379 | 65.46 | 31.72 | 19.68 | 10.59 | 10.87 | 15.21 |
| MrChr5G3389 | 16.89 | 27.15 | 13.98 | 52.85 | 38.75 | 38.23 |

**Supplementary Table 7. Primers used for detection of gene expression in Biqizhong and 2012LXRM**

| Gene | Forward primer 5' to 3' | Reverse primer 5' to 3' |
| --- | --- | --- |
| MrChr5G3291 | GGACTGGCTGTGATGGTTTT | CCAGTGTTGATCCGAGGACT |
| MrChr5G3292 | TGTCTGCTGCTACTGCAACC | ATGGAACTAACGGCATCTGG |
| MrChr5G3352 | AATTCCACCAGCATCTTTGG | CCCCTTCACTTGAAGCTCTG |
| MrChr5G3275 | AGCGAGGGAAGAGACATCAA | CCCATATGGTGAATGGTTCC |
| MrChr5G3163 | ATATGAAGGCCAGCACAACC | GCACCACCTTGGCTATTCAT |
| MrChr5G3024 | AGGACAATCCAGCAATCACC | CTTCGATCACCTCTGCTTCC |
| MrChr5G3207 | CTTTCCACGTGATCCCAAGT | TCATCGTCAGCCATTGGATA |
| MrChr5G3379 | ACTTCCACCAGGGTATGCAG | CATCCGCCATTCTCTTGATT |
| MrChr5G2994 | CTTGTCCATTGGGAACTCGT | GTTCCGGAGTCTGTGATGGT |
| MrChr5G3073 | AAGAGAAGCAAATGGGAGCA | GCCTTCAAAGCCTTCACAAA |
| MrActin | AATGGAACTGGAATGGTCAAGGC | TGCCAGATCTTCTCCATGTCATCCCA |

**Supplementary Table 8. Comparison of genetic maps between Biqizhong×2012LXRM and Biqi ×Dongkui**

| Population | Biqi ×Dongkui | Biqizhong×2012LXRM |
| --- | --- | --- |
| F1 Individual | 95 | 140 |
| Sequencing | RAD-sequencing | Re-sequencing |
| Data (Gb) | 247.64 | 902.11 |
| Markers number | 3,191 | 31,431 |
| Size (cM) | 491 | 1351.85 |
| Average (cM) | 0.12 | 0.04 |

**Supplementary Table 9. Comparison between reference genomes**

| Origin | Sequence data(Gb) | K-mer depth | Estimated genome size(Mb) | Sequence length(Mb) | Coverage (%) | Contig N50 | Number of Scaffold |
| --- | --- | --- | --- | --- | --- | --- | --- |
| Jiao et al. (2012) | 9.01 | 26X | 323 | 255.7 | 79.16 | 295bp | 273,161 |
| Ren et al. (2019) | 13.7 | 45.01X | 304.38 | 289.92 | 95.25 | 68.65kb | 1,431 |
